# Temporal patterns of alcohol use in alcohol use disorder: a 12-month ecological momentary assessment

**DOI:** 10.1007/s00406-026-02294-y

**Published:** 2026-08-04

**Authors:** Nadja S. Bahr, Andreas Heinz, Michael A. Rapp, Dominik Reichert, Markus Reichert, Paula Richter, Sarah-Lena Wagner, Sabrina Dörr, Rika Groß, Jan Luca Wiedenhöft, Johannes Steffen, Hao Chen, Bernd Lenz, Michael N. Smolka, Tobias Banaschewski, Patrick Bach, Falk Kiefer, Ulrich W. Ebner-Priemer, Gianna Spitta, Fabian Arntz

**Affiliations:** 1 Department of Psychiatry and Neurosciences, Charité-Universitätsmedizin Berlin, corporate member of Freie Universität Berlin and Humboldt-Universität zu Berlin, Berlin, Germany; 2 Department of Psychosomatic Medicine, Charité-Universitätsmedizin Berlin, Berlin, Germany; 3 German Center for Mental Health (DZPG), Partner site Berlin-Potsdam, Berlin, , Germany; 4 Department of Psychiatry and Psychotherapy, University of Tübingen, Tübingen, Germany; 5 German Center for Mental Health (DZPG), Partner site Tübingen, Tübingen, Germany; 6 Department of Psychiatry, Technische Universität Dresden, Dresden, Germany; 7 Department of Social and Preventive Medicine, University of Potsdam, Potsdam, Germany; 8 Department of eHealth and Sports Analytics, Faculty of Sports Science, Ruhr University Bochum (RUB), Bochum, Germany; 9 Department of Psychiatry and Psychotherapy, Medical Faculty Mannheim, Central Institute of Mental Health, Heidelberg University, Mannheim, Germany; 10 Department of Sport and Exercise Science, Faculty of Natural and Life Sciences, University of Salzburg, Salzburg, Austria; 11 Institute of Sports and Sports Science, Karlsruhe Institute of Technology (KIT), Karlsruhe, Germany; 12 Department of Addictive Behavior and Addiction Medicine, Medical Faculty Mannheim, Central Institute of Mental Health, Heidelberg University, Mannheim, Germany; 13 German Center for Mental Health (DZPG), Partner site Mannheim-Heidelberg-Ulm, Mannheim, , Germany; 14 Department of Child and Adolescent Psychiatry, Medical Faculty Mannheim, Central Institute of Mental Health, Heidelberg University, Mannheim, Germany

**Keywords:** Alcohol consumption, Alcohol use disorder, Ecological momentary assessment, Temporal dynamics, Seasonality, Assessment reactivity

## Abstract

Alcohol consumption (AC) is a major contributor to preventable morbidity and mortality. Fine-grained, near real-time data may capture short-term fluctuations in alcohol use, yet evidence on long-term temporal drinking patterns remains limited. Using smartphone-based ecological momentary assessment (EMA) over 12 months, we quantified weekly, seasonal, and pandemic-related drinking patterns in individuals aged 16–66 with alcohol use disorder (AUD) recruited February 2020–September 2024. Outcomes were modelled as drinking frequency (any vs. none per day) and drinking-day amount (g/day on drinking days). Mixed-effects models tested weekends, holiday-related days, annual seasonality, lockdown periods, and time since study entry. Among 670 participants, drinking frequency and drinking-day amount increased on weekends (frequency: β = 0.354; amount: β = 0.078; both *p* < .001) and holiday-related days (frequency: β = 0.319; amount: β = 0.056; both *p* < .001). During lockdowns, drinking frequency was higher (β = 0.074; *p* = .007), whereas drinking-day amount did not change. Seasonal variation was observed (frequency: β = 0.252; amount: β = 0.035; both *p* < .001), with December peaks and January declines. Drinking frequency declined early after study entry (β = −0.0005; *p* = .002) and then remained stable at a lower level than at study entry, with no time trend in drinking-day amount. Long-term EMA captured drinking patterns and suggested possible assessment-related changes early after study entry. Higher-consumption windows (weekends, holiday-related days, December) may help inform the timing of prevention and intervention strategies; causal effects of repeated EMA (i.e., assessment reactivity) should be tested in randomized controlled trials.

**Supplementary Information:**

The online version contains supplementary material available at https://doi.org/10.1007/s00406-026-02294-y.

## Introduction

Alcohol consumption (AC) is a major contributor to preventable morbidity and mortality [1–4]. Alcohol use may vary with contextual and temporal conditions, including weekly routines, seasonal periods, and changes in daily structure [5–9]. While population-level data indicate increased drinking on weekends and public holidays [7, 9, 10], as well as systematic attempts to reduce consumption during initiatives like “Dry January” [11, 12], long-term within-person temporal variation in alcohol use among individuals with alcohol use disorder (AUD) remains less well characterized. Furthermore, COVID-19 pandemic-related restrictions provide a naturalistic context to examine how shifts in routines relate to drinking patterns [13]. Evidence has been mixed, with reporting increases [14], decreases [15, 16], or otherwise heterogeneous findings in AC during the pandemic [17]. This heterogeneity may underscore the importance of distinguishing different dimensions of alcohol use (e.g., consumption volume and drinking frequency) [17]. Disentangling these dimensions across contexts and time may help clarify temporal drinking patterns and facilitate the identification of predictable higher-risk windows. Such windows may help inform the timing and targets of prevention and early intervention in alcohol use [18–21]. However, traditional retrospective assessments provide limited insight into within-person fluctuations over time, as they are prone to recall bias [22] and lack the granularity required to capture time-varying drinking patterns [23–26]. Ecological momentary assessment (EMA) can help address some of these limitations by capturing alcohol use repeatedly close in time to the event [24, 27] and may improve ecological validity by assessing these changing processes as they unfold in participants’ day-to-day lives [24]. This approach enables fine-grained modelling of within-week and seasonal rhythms. At the same time, intensive repeated EMA may introduce assessment reactivity, systematic changes in behaviour induced by the assessment itself [23, 24]. In alcohol research, such reactivity has been observed as short-term and concentrated early in monitoring in some studies [28]; however, other findings suggest generally limited reactivity, with effects that may be context-dependent [22, 29]. Moreover, to characterize drinking patterns, separating drinking frequency from drinking-day amount (intensity) may be important, as aggregate metrics can mask changes in these dimensions [30]. Therefore, to examine temporal variation in alcohol use, we used 12-month smartphone-based EMA to assess drinking frequency and drinking-day amount in daily life among participants meeting criteria for AUD. Building on prior EMA work [15, 31], this study tested four hypotheses regarding drinking frequency and drinking-day amount: (1) weekend and holiday-related increases, (2) lockdown-related shifts (Germany’s hard-lockdown period), (3) seasonal variation with December peaks and January declines, and (4) early changes after study entry (consistent with assessment reactivity). Potential moderation of these hypothesized temporal patterns in drinking frequency and drinking-day amount by age and gender was explored.

## Methods

### Study design

This prospective cohort study was conducted within the Collaborative Research Centre TRR265 on substance use disorders [32]. Related analyses from the same TRR265 cohort framework have been reported previously, based on overlapping subsamples [15, 31]. The present study focuses on the complete sample. Individuals were recruited between February 2020 and September 2024 at three German academic sites (Charité – Universitätsmedizin Berlin, Technical University Dresden, Central Institute of Mental Health Mannheim). Participants were recruited online and through advertisements and flyers at the three study sites, outside clinical treatment settings. A telephone screening was performed to verify inclusion and exclusion criteria. Participants completed baseline assessments, diagnostic interviews, and ecological momentary assessment (EMA) over 12 months. The study was approved by the local ethics committees responsible for the participating sites, and all participants provided written informed consent. For participants younger than 18 years, informed consent was co-signed by a parent or legal guardian. Participants received modest financial compensation for completing the EMA surveys, in accordance with local ethics committee approvals. Reporting followed the STROBE guidelines for observational studies.

### Participants

Of 3492 individuals screened, 749 met the inclusion criteria and 670 completed EMA assessments. Eligible participants were aged 16–66 years and met 2–10 criteria for alcohol use disorder according to the Diagnostic and Statistical Manual of Mental Disorders, Fifth Edition (DSM-5) [33], but did not require medically supervised alcohol withdrawal and reported no intention to seek treatment. Participants were required to have sufficient German proficiency and agree to use an Android smartphone. Exclusion criteria were current use of drugs or medications affecting the central nervous system; contraindications to magnetic resonance imaging (MRI); pregnancy or breastfeeding; medical history of DSM-5 bipolar disorder, psychotic disorder, schizophrenia or schizophrenia spectrum disorder, or substance use disorders other than alcohol, nicotine, or cannabis; and medical history of severe head injury or other severe central nervous system disorders.

### Measures

#### Baseline assessments

AUD symptom counts were derived from the German version of the Structured Clinical Interview for DSM-5 Disorders – Clinician Version (SCID-5-CV; [34]) to confirm DSM-5 criteria and quantify severity for research classification. Interviews were conducted by a team of trained staff, including psychologists and research assistants, who underwent standardized SCID training. To ensure diagnostic reliability, ambiguous cases were discussed within an interdisciplinary clinical team until a consensus was reached. Sociodemographic and self-report data were collected at baseline (e.g., age, self-reported gender, and education); gender was coded as women or men for analyses.

#### Ecological momentary assessment (EMA)

Participants completed 12 months of EMA using e-diaries via a smartphone application (movisensXS; movisens GmbH, Karlsruhe, Germany) that complies with the EU General Data Protection Regulation (GDPR) and the Berlin Data Protection Act (Berliner Datenschutzgesetz; BlnDSG). They received daily prompts at 12 PM (optional deferral until 8 PM; up to 5 reminders). At each prompt, participants reported AC for up to the previous 2 days, specifying beverage type and volume. Study staff supported app installation and initial entries; Android devices were provided if needed.

### Outcomes

Outcome measures were based on EMA-reported AC. Primary outcomes were (1) drinking frequency (any vs. none per day) and (2) drinking-day amount (g/day on drinking days). Overall daily AC (overall AC; g/day) was derived from these measures and is reported for comparability in Online Resource 1.

### Pandemic context

EMA data collection spanned both non-pandemic and COVID-19 periods and included Germany’s “light” and “hard” lockdown phases: pre-lockdown (October 2–November 1, 2020), light lockdown (November 2–December 15, 2020), and hard lockdown (December 16, 2020–February 28, 2021) [35]. For analysis, we operationalized lockdown exposure as a binary indicator contrasting hard-lockdown days with all other assessment days (i.e., pre-lockdown, light-lockdown, and non-lockdown days outside the lockdown period), reflecting the most stringent restriction phase.

### Statistical analysis

Separate multilevel models were estimated for drinking frequency and drinking-day amount. Predictors represented weekly (H1a), holiday-related (H1b), and holiday-by-weekend interaction (H1c) effects, as well as lockdown (H2), seasonal (H3), and time-since-study-entry (H4) effects. Linear mixed models were used for drinking-day amount, logistic mixed models for drinking frequency. Drinking-day amount was log-transformed due to right-skewness; coefficients for this outcome are reported on the log scale. Exponentiating coefficients yields multiplicative effects on the grams scale. Level-1 predictors captured within-person variation (weekday/weekend, holiday-related days, lockdown phase, sine-transformed day-of-year function, and calendar month contrasts for December and January). Level-2 predictors (age, gender, age × gender) captured between-person differences. Weekend (Friday–Saturday) and holiday-related day indicators tested H1 effects; their interaction examined amplified holiday peaks. Holiday-related days were defined as public holidays and the day preceding a public holiday. A binary indicator for the hard lockdown tested H2 and its interactions with weekend and month contrasts. Seasonal effects (H3) were modelled with December/January contrasts and a sine-wave term (centred on September 7). Time-since-study-entry effects (H4) were modelled using a third-order polynomial to capture potential systematic changes over monitoring time. Continuous predictors were person-mean centred and categorical variables were coded using sequential-difference coding. Model fit guided retention of random slopes based on Akaike and Bayesian Information Criteria [36]. All models were estimated in R (lme4 package) using maximum likelihood estimation with α = 0.05. Results are reported as regression coefficients (β) with standard errors (SEs) and P values. Missing EMA entries were handled in the mixed models via maximum likelihood, assuming data were missing at random conditional on observed covariates and random effects. No imputation was applied. Full model specifications are provided in Online Resource 1 (eAppendix 1).

## Results

Of 670 participants (261 women [39.0%]; mean [SD] age, 37.4 [12.7] years), all met 2–10 DSM-5 criteria for AUD. Participant characteristics and EMA compliance/descriptive statistics are presented in Table 1. Participants completed 12 months of EMA, yielding AC data for a median of 5.83 days per week (IQR, 3.7–6.8) and an overall mean response rate of 68% (67.5% in women; 71.2% in men).

**Table 1:** Participant characteristics and EMA compliance/descriptive statistics (*N* = 670)

Characteristic	Finding
Age, mean (SD) [range]	37.4 (12.7) [16–66]
Gender, No. (%)	
Women	261 (39.0%)
Men	409 (61.0%)
AUD criteria, mean (SD) [range]	4.14 (1.66) [2–10]
AUD criteria, No. (%)	
2	128 (19.1%)
3	162 (24.2%)
4	130 (19.4%)
5	107 (16.0%)
6	74 (11.0%)
7	50 (7.5%)
8	12 (1.8%)
9	6 (0.9%)
10	1 (0.1%)
Education level, No. (%)	
Low (≤ 9 years)	33 (4.9%)
Medium (10–12 years)	122 (18.2%)
High (university-access)	477 (71.2%)
Missing	38 (5.7%)
EMA compliance	
Days with AC data per week, median (IQR)	5.83 (3.7–6.8)
EMA prompt response rate, %	68.0
Overall daily AC, mean (SD), g/day	34.9 (24.7)

*AC* alcohol consumption, *AUD* alcohol use disorder, *EMA* ecological momentary assessment, *IQR* interquartile range, *SD* standard deviation

**Fig. 1:**
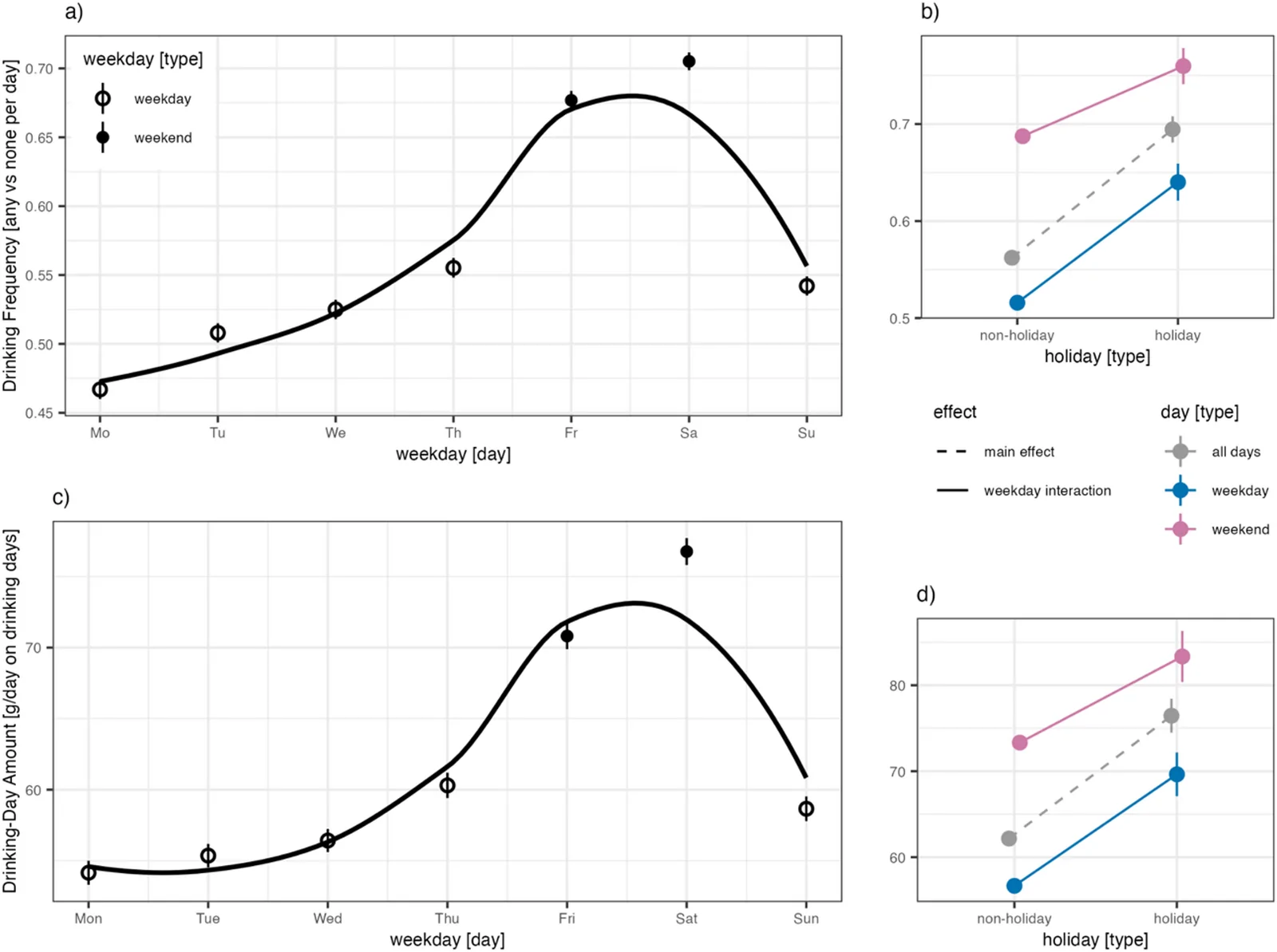
Model-implied means of drinking frequency and drinking-day amount by day of week and holiday status (H1). **a** Drinking frequency (any vs. none per day) by day type (weekday vs. weekend); **b** Drinking frequency (any vs. none per day) comparing holiday-related vs. non–holiday-related days overall and by day type (weekday vs. weekend); **c** Drinking-day amount (g/day on drinking days) by day type (weekday vs. weekend); **d** Drinking-day amount (g/day on drinking days) comparing holiday-related vs. non–holiday-related days overall and by day type (weekday vs. weekend); Weekend was defined as Friday–Saturday (vs. all other days); Points indicate model-implied means

In line with our first hypothesis (H1a), drinking frequency (β = 0.354; SE = 0.030; *p* < .001) and drinking-day amount (β = 0.078; SE = 0.009; *p* < .001) were significantly higher on weekends (Friday–Saturday) than on weekdays (Fig. 1a, c). Consistent with H1b, both drinking frequency (β = 0.319; SE = 0.035; *p* < .001) and drinking-day amount (β = 0.056; SE = 0.011; *p* < .001) were higher on holiday-related days (Fig. 1b, d). A significant holiday-related day × weekday type (weekday vs. weekend) interaction (H1c) indicated that the holiday-related increase was attenuated on weekends relative to weekdays (Fig. 1b, d), for both drinking frequency (β = − 0.135; SE = 0.022; *p* < .001) and drinking-day amount (β = − 0.050; SE = 0.006; *p* < .001).

All fixed effects, including main and interaction terms and non–statistically significant estimates, for drinking frequency and drinking-day amount are summarized in Table 2. Results for overall daily AC are reported in Online Resource 1 (eTable 1). Consistent with our second hypothesis (H2), during hard lockdown, drinking frequency increased (β = 0.074; SE = 0.027; *p* = .007), whereas drinking-day amount remained unchanged (β = − 0.007; SE = 0.007; *p* = .36). Model-implied means by lockdown status and calendar time are provided in Online Resource 1 (eFigure 1 a–c).

**Fig. 2:**
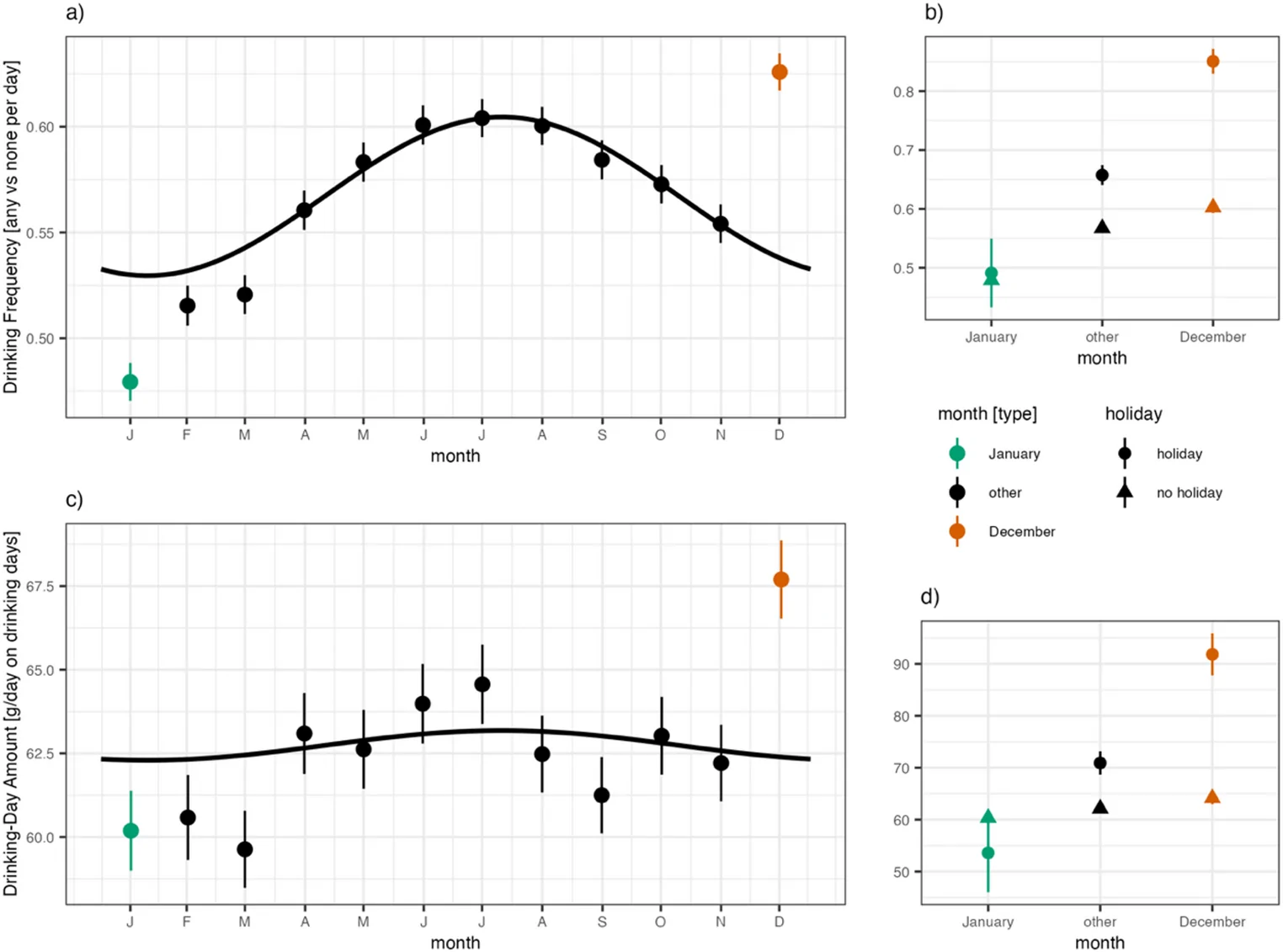
Seasonal variation in drinking frequency and drinking-day amount (H3). **a** Drinking frequency (any vs. none per day) across the year, illustrating higher estimates in December and lower estimates in January; **b** Drinking frequency comparing holiday-related days vs. non–holiday-related days in January, December, and other months; **c** Drinking-day amount (g/day on drinking days) across the year, illustrating higher estimates in December and lower estimates in January; **d** Drinking-day amount comparing holiday-related days vs. non–holiday-related days in January, December, and other months; Holiday-related days include public holidays and the day preceding a public holiday

**Fig. 3:**
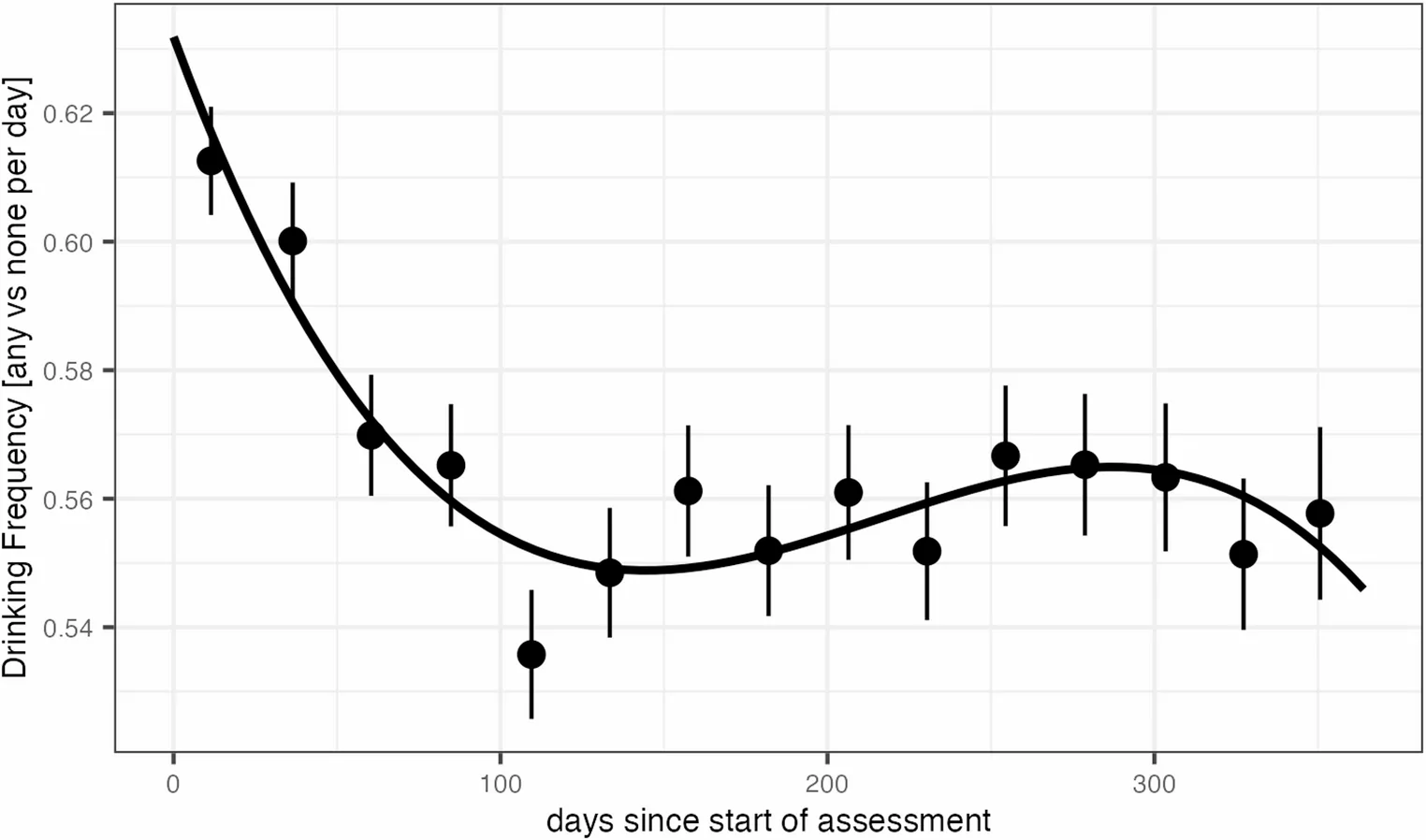
Early changes in drinking frequency after study entry (H4). Modelled estimates of drinking frequency (any vs. none per day) across the 12-month EMA period. Estimates showed an early decline over approximately the first 100 days of monitoring, followed by relatively stable levels thereafter

Seasonal effects consistent with our third hypothesis (H3) were pronounced: December was associated with higher drinking frequency (β = 1.168; SE = 0.076; *p* < .001) and greater drinking-day amount (β = 0.184; SE = 0.019; *p* < .001), while January showed declines (drinking frequency: β = − 0.377; SE = 0.104; *p* < .001; drinking-day amount: β = − 0.135; SE = 0.032; *p* < .001; Fig. 2a–d). The sine-transformed day-of-year function supported a cyclical annual pattern, with significant effects for drinking frequency (β = 0.252; SE = 0.029; *p* < .001) and drinking-day amount (β = 0.035; SE = 0.008; *p* < .001; Fig. 2a, c). December showed an additional elevation beyond the sinusoidal annual term (Fig. 2b, d). Consistent with our fourth hypothesis (H4), significant effects of time since study entry emerged for drinking frequency but not drinking-day amount. Drinking frequency declined early after study entry (linear β = − 0.0005; SE = 0.0002; *p* = .002), and the higher-order time terms were also significant (quadratic *p* < .001; cubic *p* < .001; Fig. 3). Drinking-day amount showed no evidence of a time trend (linear *p* = .73; quadratic *p* = .23; cubic *p* = .20).

**Table 2:** Fixed effects of predictors on drinking frequency and drinking-day amount

Predictor	Drinking frequency				Drinking-day amount			
	β	SE	z	*p* value	β	SE	z	*p* value
Intercept	0.9802	0.0756	12.96	**< 0.001**	3.8601	0.0213	181.11	**< 0.001**
Age (cohort)	0.0522	0.0050	10.37	**< 0.001**	−0.0003	0.0014	−0.25	0.80
Gender (men)	0.1269	0.0646	1.97	**0.05**	0.1746	0.0174	10.03	**< 0.001**
Weekend (Friday–Saturday vs. other days)	0.3537	0.0297	11.90	**< 0.001**	0.0779	0.0086	9.03	**< 0.001**
Holiday-related day (vs. other days)	0.3193	0.0348	9.18	**< 0.001**	0.0557	0.0113	4.92	**< 0.001**
Hard lockdown (vs. other days)	0.0739	0.0272	2.72	**0.007**	−0.0067	0.0073	−0.91	0.36
Annual pattern (sine)	0.2518	0.0290	8.67	**< 0.001**	0.0350	0.0080	4.37	**< 0.001**
January (“Dry January”)	−0.3765	0.1037	−3.63	**< 0.001**	−0.1351	0.0318	−4.25	**< 0.001**
December	1.1682	0.0757	15.42	**< 0.001**	0.1838	0.0189	9.71	**< 0.001**
Observation period, linear	−0.0005	0.0002	−3.11	**0.002**	0.0000	0.0001	0.34	0.73
Observation period, quadratic	0.0000	0.0000	9.08	**< 0.001**	0.0000	0.0000	1.19	0.23
Observation period, cubic	−0.0000	0.0000	−7.04	**< 0.001**	−0.0000	0.0000	−1.29	0.20
Age × gender	0.0028	0.0050	0.56	0.58	0.0003	0.0013	0.25	0.81
Weekend × hard lockdown	−0.0134	0.0170	−0.79	0.43	−0.0201	0.0051	−3.93	**< 0.001**
Holiday-related day × weekend	−0.1350	0.0220	−6.13	**< 0.001**	−0.0495	0.0064	−7.74	**< 0.001**
Weekend × age	−0.0060	0.0012	−5.04	**< 0.001**	−0.0033	0.0004	−9.37	**< 0.001**
January × hard lockdown	0.0551	0.0593	0.93	0.35	−0.0191	0.0140	−1.36	0.17
December × hard lockdown	0.2808	0.0488	5.76	**< 0.001**	0.0180	0.0140	1.28	0.20
January × holiday-related day	−0.1327	0.0845	−1.57	0.12	−0.1193	0.0298	−4.00	**< 0.001**
December × holiday-related day	0.5105	0.0585	8.73	**< 0.001**	0.0905	0.0142	6.36	**< 0.001**

Drinking frequency was modelled using a mixed-effects logistic regression (β estimates on the log-odds scale). Drinking-day amount was modelled using a mixed-effects linear model on drinking days (β on the model scale). Weekend days were Friday–Saturday; holiday-related days included public holidays and the preceding day; lockdown contrasted hard-lockdown days with all other assessment days

*SE* standard error, *β* fixed-effect estimate. Drinking frequency was defined as any vs. none per day; drinking-day amount as grams of alcohol per day on drinking days

**Fig. 4:**
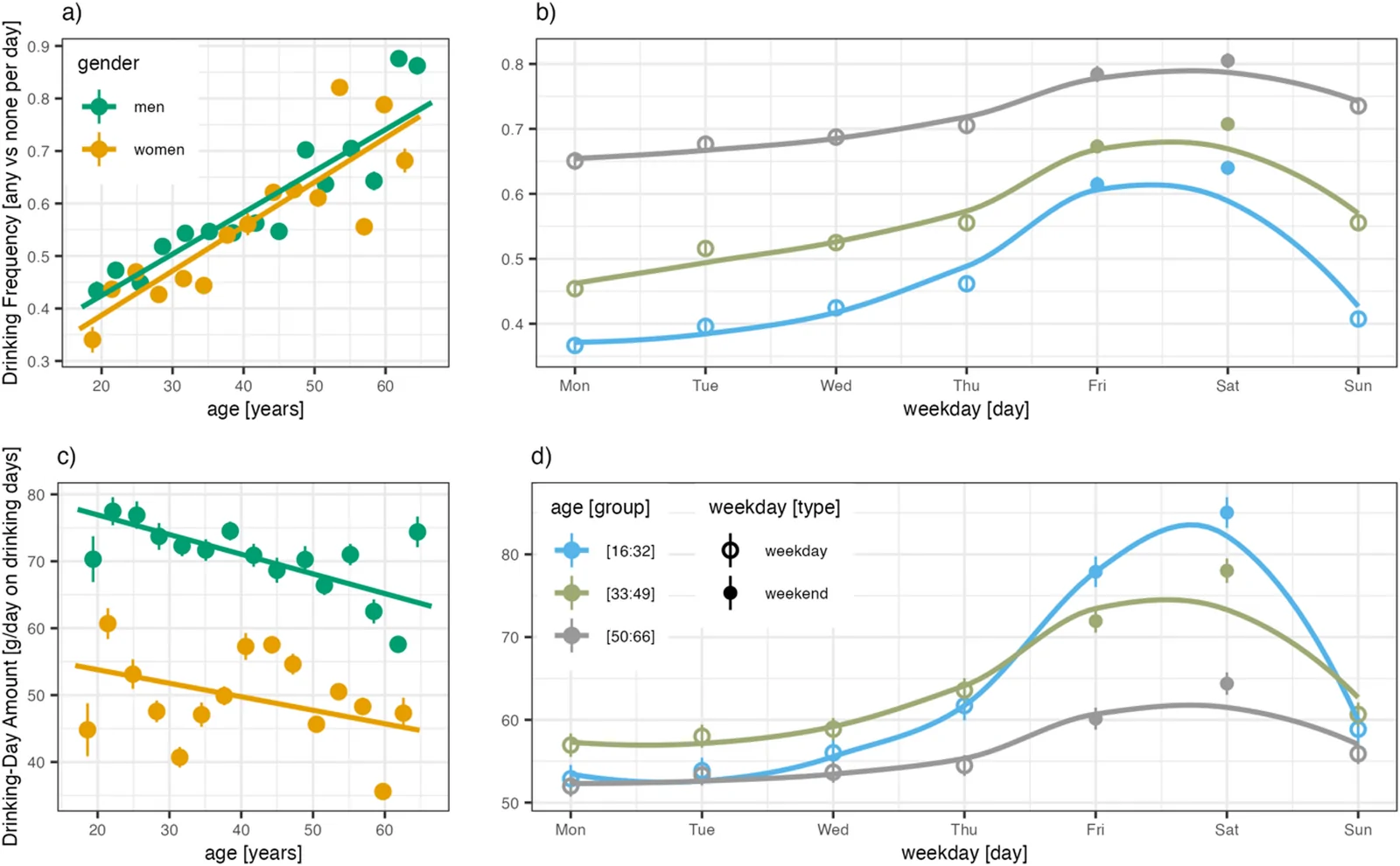
Associations of age, gender, and day type with drinking frequency and drinking-day amount. **a** Association of age with drinking frequency (any vs. none per day), by gender; **b** Day-of-week variation in drinking frequency (any vs. none per day), by age group; **c** Association of age with drinking-day amount (g/day on drinking days), by gender; **d** Day-of-week variation in drinking-day amount (g/day on drinking days), by age group. Weekend was defined as Friday–Saturday (vs. all other days). Drinking-day amount is presented on the grams scale (model-based estimates back-transformed from the log scale). Age groups were defined as 16–32, 33–49, and 50–66 years

Demographic effects were observed for age and gender. Older participants reported higher drinking frequency (β = 0.052; SE = 0.005; *p* < .001) but no age difference in drinking-day amount (β = − 0.0003; SE = 0.0014; *p* = .80). Men showed slightly higher drinking frequency (β = 0.127; SE = 0.065; *p* = .05) and greater drinking-day amounts (β = 0.175; SE = 0.017; *p* < .001). No age × gender interactions were observed (drinking frequency: β = 0.0028; SE = 0.0050; *p* = .58; drinking-day amount: β = 0.0003; SE = 0.0013; *p* = .81; Fig. 4a, c). Age moderated weekend effects: older individuals showed weaker weekend increases in drinking frequency (β = − 0.006; SE = 0.001; *p* < .001) and drinking-day amount (β = − 0.003; SE = 0.0004; *p* < .001; Fig. 4b, d).

Additional interaction effects involving lockdown status and holiday-related temporal contrasts further refined these patterns. For drinking frequency, the December increase remained evident during hard lockdown (β = 0.281; SE = 0.049; *p* < .001), whereas no corresponding December effect was observed for drinking-day amount (β = 0.018; SE = 0.014; *p* = .20). Weekend-related increases were attenuated during hard lockdown for drinking-day amount (β = − 0.020; SE = 0.005; *p* < .001). No significant change was observed for drinking frequency (β = − 0.013; SE = 0.017; *p* = .43). On holiday-related days in January, drinking-day amount decreased (β = − 0.119; SE = 0.030; *p* < .001); the corresponding effect for drinking frequency was not significant (β = − 0.133; SE = 0.085; *p* = .12). No significant interactions emerged between January reductions and lockdown restrictions (drinking frequency: β = 0.055; SE = 0.059; *p* = .35; drinking-day amount: β = − 0.019; SE = 0.014; *p* = .17). The December × holiday-related day interaction indicated further increases in drinking frequency (β = 0.511; SE = 0.059; *p* < .001) and drinking-day amount (β = 0.091; SE = 0.014; *p* < .001).

Random-effects estimates indicated considerable interindividual variability in temporal slopes for weekend, holiday-related day, annual seasonality, and December effects. Random-effects variance components and correlations for all drinking outcomes are reported in Online Resource 1 (eTable 2a–c), and correlations between individual-specific effects are shown in Online Resource 1 (eFigure 2a–c). Correlations among random slopes (i.e., correlations between participant-specific random effects) for drinking frequency and drinking-day amount suggested co-occurrence of stronger effects: participants with stronger weekend effects also showed stronger holiday-related day increases (*r* = .64 for drinking frequency; eTable 2b and eFigure 2a; *r* = .53 for drinking-day amount; eTable 2c and eFigure 2c). In addition, participants with stronger annual seasonality showed larger December increases (*r* = .50 for drinking frequency; eTable 2b and eFigure 2b; *r* = .29 for drinking-day amount; eTable 2c). Corresponding results for overall daily AC are provided in Online Resource 1 (eTable 2a).

## Discussion

This study provides yearlong EMA data on drinking frequency and drinking-day amount in individuals with AUD (*N* = 670). Our findings showed pronounced weekend and holiday-related peaks (H1), lockdown-related differences (H2), and seasonal fluctuations (H3) including December increases and early-year declines. Possible assessment-related changes after study entry were also observed (H4). Lockdown-related differences (H2) and assessment-related changes (H4) occurred for drinking frequency but not drinking-day amount. These findings extend prior EMA work documenting real-world temporal and pandemic-related variation in AUD [15, 31]. Our results are consistent with population-level evidence of greater weekend consumption [9, 10] and winter-holiday peaks [7, 10]. In line with H1, both frequency and amount increased on weekends and holidays, suggesting that drinking in AUD may remain synchronized with temporal rhythms, mirroring prior analyses of this cohort [15, 31]. This pattern may be compatible with habitual and goal-directed influences rather than purely compulsive use [37], highlighting weekends and holidays as potential higher-risk windows for time-targeted intervention [15]. Consistent with H2, our findings align with systematic reviews indicating heterogeneous pandemic-related changes in alcohol use across individuals and countries [6, 17]. In our data, during Germany’s hard lockdown (December 16, 2020–February 28, 2021) drinking frequency increased while drinking-day amount remained unchanged. Additionally, the weekend-related increase in drinking-day amount was attenuated (i.e., a flatter weekly pattern), whereas the weekend-related increase in frequency showed no change. A reduced weekday–weekend contrast during hard lockdown was also reported in prior analyses of this cohort [15]. Together, these results suggest that drinking in AUD varies with lockdown-related disruptions of routines, including a reduced weekday–weekend contrast [15]. These findings may inform monitoring by highlighting windows of variability (e.g., weekends) and contextual factors (e.g., changes in work/leisure structure or social opportunities) linked to heterogeneous pandemic-related changes, including increased drinking in some subgroups [6]. Prior analyses reported lower aggregate consumption during lockdown [15, 31]; separating drinking frequency from drinking-day amount may therefore provide additional insight beyond aggregate measures [30]. Consistent with H3, we observed seasonal variations: December increases and early-year declines. This aligns with population studies [7] and “Dry January” trends [11, 12]. The December increase may reflect the high density of social and cultural occasions [10], while early-year declines may be contextualized by temporary abstinence efforts [11, 12]. Our EMA data suggest that these patterns may also be present in individuals with AUD, where drinking patterns may fluctuate in synchrony with broader calendar-based rhythms. Prevention and intervention efforts may benefit from targeting these annual higher-risk periods [7]. Consistent with H4, a decline in drinking frequency over the first three months, followed by stabilization below baseline, was observed, while drinking-day amount showed no evidence of change over time. Assessment reactivity or self-monitoring–related change may explain early declines [24], though diary studies suggest reactivity can be short-lived [28]. Indeed, repeated self-monitoring has been associated with small reductions in alcohol use in app-based daily monitoring studies, particularly among hazardous drinkers [38]. One possible explanation is assessment reactivity: close temporal proximity between assessment and behaviour may prompt momentary reflection in some participants [24]. This makes reactivity a relevant consideration for AUD cohorts. The subsequent stabilization of drinking frequency in our data may align with evidence that participants eventually habituate to the monitoring protocol, leading to a decay of the initial reactivity effect [24]. However, self-monitoring effects may be short-lived, and additional intervention components beyond assessment may be needed for sustained change [28, 39]. Furthermore, men consumed higher amounts, consistent with broader evidence of persistent gender differences [1]. However, EMA work suggests these differences also involve mood- and stress-related correlates [31] and evolving sex differences [40]. Older participants reported higher frequency but similar drinking-day amounts, indicating age variations in regularity rather than quantity. Age moderated weekend effects, with higher age linked to less weekend-concentrated drinking, suggesting that changing drinking patterns in later life may be shaped by social and structural context (e.g., partnership, health status, and socio-economic position) [41].

### Clinical and public health implications

Our results identify higher-risk windows (e.g., weekends, holiday-related periods, December peaks) that may be considered intervention targets. Brief interventions before high-risk windows could address short-term increases in drinking frequency or amount. This aligns with proposed prior-to-onset JITAI triggering [20], and EMA evidence emphasizing assessment timing in close proximity to use-related events [42]. Initiatives like “Dry January”, which encourage temporary abstinence may reflect the concept of temporally bounded behaviour-change efforts [43]. Our findings highlight pronounced higher-risk windows, suggesting that daily and seasonal fluctuations in drinking frequency and drinking-day amount may help inform adaptive, context-sensitive prevention [44]. These patterns may be relevant for just-in-time adaptive intervention (JITAI) models [45, 46]. For example, prompts could be scheduled around periods of higher drinking, such as weekends or holidays. Embedding prompts in everyday devices could enable delivery of timely messages in everyday life [47]. Beyond assessment, such approaches may benefit from additional intervention components, including tailored or automated feedback (e.g., graphical feedback on behaviour patterns) [28], skills-based or just-in-time elements, including just-in-time support during high-risk situations [28, 39], and in vivo skills training [39]. Aligning intervention timing with calendar rhythms may improve the fit with real-world patterns and increase ecological validity, a hypothesis to be tested in future studies [24, 27]. Overall, smartphone-delivered momentary interventions may leverage higher-risk windows; efficacy should be evaluated in controlled trials.

### Methodological implications

Explicitly modelling calendar time in EMA analyses may be important to account for weekday, monthly, and seasonal variation in both drinking frequency and drinking-day amount. Assessment reactivity could be evaluated to characterize habituation and other time-on-study changes associated with repeated assessments, which may depend on sampling protocol and individual characteristics [48]. Further work is needed to determine whether such changes introduce bias. Because these changes may not be confined to the earliest study phase, modelling time since study entry with flexible, non-linear terms may help distinguish transient adaptation from sustained change. Future studies could examine how assessment frequency and feedback interact with participant characteristics and context. These processes should be evaluated in larger, diverse cohorts using standardized reporting frameworks and multimodal EMA designs that integrate passive sensing [49].

### Limitations

Participants were recruited through advertisements, yielding a convenience sample (mild to severe AUD).

This recruitment may have introduced self-selection bias, favouring individuals more motivated or able to participate in intensive monitoring, which may have influenced the observed reductions. Apparent reductions might also reflect regression to the mean given the elevated baseline use. Outcome data were self-reported and may be affected by misreporting (e.g., social desirability or recall difficulties), potentially affecting estimates of peak drinking days. To address potential nonresponse (compliance) bias due to systematic missingness, we tested whether individuals with heavier alcohol use or lower daily structure responded less consistently; compliance rates showed no clear association with drinking frequency or drinking-day amount, suggesting limited evidence for participant-level compliance bias. We also tested day-level differential nonresponse and found slightly higher missingness on Fridays (+ 6%) and, when aggregating weekend days (Friday–Saturday), ~ 3% higher missingness compared with Mondays. These differences were statistically significant but small in magnitude, suggesting limited day-level bias. Missing data were addressed using maximum likelihood estimation, which assumes that missingness depends on observed but not unobserved factors; while this approach reduces bias, residual effects of selective nonresponse cannot be fully ruled out. The naturalistic design and lack of a control group preclude causal inference. Moreover, as the study spanned 2020 to 2024, the findings may reflect a period of significant transition from pandemic-related restrictions to standard, non-pandemic routines. These temporal patterns should be considered as descriptive of this specific historical context. As in other EMA research, day-level changes could be driven by unmeasured factors, which should be taken into account when interpreting the observed patterns. Future research should evaluate these findings in more diverse cohorts to test whether these patterns generalize beyond this transitional period.

## Conclusion

Using 12-month smartphone-based EMA, we quantified weekly, seasonal, and pandemic-related drinking dynamics in individuals with AUD, separately modelling drinking frequency (any vs. none per day) and drinking-day amount (g/day on drinking days). We observed pronounced increases in both outcomes on weekends and holiday-related days. During Germany’s hard lockdown period, drinking frequency was higher, whereas drinking-day amount did not change. We also observed annual seasonal variation in both outcomes, with December peaks and early-year (January) declines. Finally, drinking frequency showed an early decline after study entry followed by stabilization at a lower level than at study entry, while drinking-day amount showed no evidence of a time trend. These findings highlight temporally bounded higher-risk windows that may help inform the timing of prevention and intervention strategies aligned with calendar-based rhythms. The early decline in drinking frequency is compatible with short-lived assessment reactivity and/or self-monitoring effects; however, causal effects require controlled trials, and sustained behaviour change may depend on additional components beyond assessment alone, such as tailored or automated feedback and skills-based or just-in-time intervention elements. Ultimately, these temporal patterns underscore EMA’s potential both as a methodological tool and as a basis for developing and testing tailored interventions.

## Supplementary Information

Below is the link to the electronic supplementary material.

Supplementary Material 1

## Data Availability

The individual-level study data are not publicly available due to ethical and privacy restrictions. Rendered analysis scripts and reports are publicly available via the Open Science Framework (OSF) repository (https://osf.io/n3zph).

## Declarations

### Compensation

Participants received monetary compensation for participation, in accordance with local ethics committee approvals.
