# Supplementary material for: Temporal patterns of alcohol use in alcohol use disorder: a 12-month ecological momentary assessment
Source: Eur Arch Psychiatry Clin Neurosci. 2026 Aug 4;276(7):3367–78. doi: 10.1007/s00406-026-02294-y (PMC13562098; doi:10.1007/s00406-026-02294-y)
Supplement: Supplementary file 1 [file 406_2026_2294_MOESM1_ESM.pdf]

Online Resource 1

Article Title:

Temporal Patterns of Alcohol Use in Alcohol Use Disorder: A 12-Month Ecological Momentary Assessment

Journal Name:

European Archives of Psychiatry and Clinical Neuroscience

Author Names:

Nadja S. Bahr, MSc[1,2,3]; Andreas Heinz, MD, PhD[4,5,6]; Michael A. Rapp, MD, PhD[3,7]; Dominik Reichert, MSc[8]; Markus Reichert, PhD[9,10]; Paula Richter, MSc[1]; Sarah-Lena Wagner, BSc[1]; Sabrina Dörr, MSc[1]; Rika Groß, MSc[9,11]; Jan Luca Wiedenhöft, MSc[7]; Johannes Steffen, MSc[6]; Hao Chen, PhD[6]; Bernd Lenz, MD[12,13]; Michael N. Smolka, MD[6]; Tobias Banaschewski, MD, PhD[13,14;] Patrick Bach, MD, PhD[12,13]; Falk Kiefer, MD[12,13]; Ulrich W. Ebner-Priemer, PhD[9,11,13]; Gianna Spitta, MD*[1,3]; Fabian Arntz, PhD*[3,7]

Affiliations:

1 Department of Psychiatry and Neurosciences, Charité-Universitätsmedizin Berlin, corporate member of Freie Universität Berlin and Humboldt-Universität zu Berlin, Berlin, Germany

2 Department of Psychosomatic Medicine, Charité-Universitätsmedizin Berlin, Berlin, Germany

3 German Center for Mental Health (DZPG), site Berlin-Potsdam, Germany

4 Department of Psychiatry and Psychotherapy, University of Tübingen, Tübingen, Germany

5 German Center for Mental Health (DZPG), partner site Tübingen, Germany

6 Department of Psychiatry, Technische Universität Dresden, Dresden, Germany

7 Department of Social and Preventive Medicine, University of Potsdam, Potsdam, Germany

8 Department of eHealth and Sports Analytics, Faculty of Sports Science, Ruhr University Bochum (RUB), Bochum, Germany

9 Department of Psychiatry and Psychotherapy, Central Institute of Mental Health, Medical Faculty Mannheim, Heidelberg University, Mannheim, Germany

10 Department of Sport and Exercise Science, Faculty of Natural and Life Sciences, University of Salzburg, Salzburg, Austria

11 Institute of Sports and Sports Science, Karlsruhe Institute of Technology (KIT), Karlsruhe, Germany

12 Department of Addictive Behavior and Addiction Medicine, Central Institute of Mental Health, Medical Faculty Mannheim, Heidelberg University, Mannheim, Germany

13 German Center for Mental Health (DZPG), partner site Mannheim-Heidelberg-Ulm, Germany

14 Department of Child and Adolescent Psychiatry, Central Institute of Mental Health, Medical Faculty Mannheim, Heidelberg University, Mannheim, Germany

Corresponding Author:

Andreas Heinz, Department of Psychiatry and Psychotherapy, University of Tübingen, Tübingen, Germany

E-mail: andreas.heinz@med.uni-tuebingen.de

Table of Contents

eAppendix 3

eAppendix 1. Full model specifications 3

eTables 6

eTable 1. Fixed effects for overall daily alcohol consumption (overall AC) 6

eTable 2. Random effects variance components and correlations of (a) Overall daily alcohol consumption (overall AC), (b) Drinking frequency (any vs none per day) and (c) Drinking-day amount (g/day on drinking days) 7

eFigures 9

eFigure 1. Model-implied means of drinking outcomes by lockdown status and calendar time 9

eFigure 2. Correlations between participant-specific random effects across temporal predictors 10

eReferences 10

eAppendix

eAppendix 1. Full model specifications

Outcomes

We estimated separate multilevel models for:

Overall daily alcohol consumption (overall AC)

Y_ij^((O)) denotes overall daily alcohol consumption (g/day) on day i for participant j. Overall AC was modelled on the log scale; coefficients for this outcome are reported on the log scale.

Drinking frequency

$Y_{ij}^{(F)} \in \{0,1\}$ indicates any drinking (1) versus none (0) on day i for participant j.

Drinking-day amount

$Y_{ij}^{(A)}$ denotes drinking-day amount on day i for participant j. Drinking-day amount (g/day on drinking days) was log-transformed due to right-skewness; coefficients for this outcome are reported on the log scale.

Level-1 predictors (within-person)

Let the following day-level predictors be defined for participant j on day i:

Weekend: indicator for Friday–Saturday versus all other days (coded as described in the main text).

Holiday-related day: indicator for public holidays and the day preceding a public holiday.

Lockdown: indicator for hard-lockdown days (Dec 16, 2020 to Feb 28, 2021) versus all other days.

Dry January: January contrast.

Drinking December: December contrast.

Annual sine: sine-transformed day-of-year function (centered as specified in the main text).

Assessment day: time since study entry modelled as a third-order polynomial.

Level-2 predictors (between-person)

Age, Gender, and Gender × Age.

Continuous predictors were person-mean centered; categorical variables were coded using sequential-difference coding.

Model 1. Drinking frequency (logistic mixed model)

$$\begin{aligned}
\operatorname{logit}(P(Y_{ij}^{(F)} = 1)) \\
&= \beta_0 + \beta_1 \text{ Weekend}_{ij} + \beta_2 \text{ HolidayRelated}_{ij} + \beta_3 \text{ Lockdown}_{ij} + \beta_4 \text{ DryJanuary}_{ij} \\
&+ \beta_5 \text{ DrinkingDecember}_{ij} + \beta_6 \text{ AnnualSine}_{ij} + \beta_7 \text{ AssessmentDay}_{ij} + \beta_8 \text{ AssessmentDay}_{ij}^2 \\
&+ \beta_9 \text{ AssessmentDay}_{ij}^3 \\
+\beta_{10} \text{ Gender}_j + \beta_{11} \text{ Age}_j + \beta_{12} (\text{Gender} \times \text{Age})_j \\
+\beta_{13} (\text{Weekend} \times \text{HolidayRelated})_{ij} + \beta_{14} (\text{Weekend} \times \text{Age})_{ij} + \beta_{15} (\text{Weekend} \times \text{Lockdown})_{ij} \\
&+ \beta_{16} (\text{DryJanuary} \times \text{Lockdown})_{ij} + \beta_{17} (\text{DrinkingDecember} \times \text{Lockdown})_{ij} \\
&+ \beta_{18} (\text{DryJanuary} \times \text{HolidayRelated})_{ij} + \beta_{19} (\text{DrinkingDecember} \times \text{HolidayRelated})_{ij} \\
+u_{0j}^{(F)} + u_{1j}^{(F)} \text{ Weekend}_{ij} + u_{2j}^{(F)} \text{ HolidayRelated}_{ij} + u_{3j}^{(F)} \text{ AnnualSine}_{ij} + u_{4j}^{(F)} \text{ DryJanuary}_{ij} \\
&+ u_{5j}^{(F)} \text{ DrinkingDecember}_{ij}.
\end{aligned}$$

Model 2. Drinking-day amount (linear mixed model; log scale)

$$\begin{aligned}
Y_{ij}^{(A)} &= \beta_0 + \beta_1 \, Weekend_{ij} + \beta_2 \, HolidayRelated_{ij} + \beta_3 \, Lockdown_{ij} + \beta_4 \, DryJanuary_{ij} \\
&+ \beta_5 \, DrinkingDecember_{ij} + \beta_6 \, AnnualSine_{ij} + \beta_7 \, AssessmentDay_{ij} + \beta_8 \, AssessmentDay_{ij}^2 \\
&+ \beta_9 \, AssessmentDay_{ij}^3 \\
+\beta_{10} \, Gender_j + \beta_{11} \, Age_j + \beta_{12} \, (Gender \times Age)_j \\
+\beta_{13} \, (Weekend \times HolidayRelated)_{ij} + \beta_{14} \, (Weekend \times Age)_{ij} + \beta_{15} \, (Weekend \times Lockdown)_{ij} \\
&+ \beta_{16} \, (DryJanuary \times Lockdown)_{ij} + \beta_{17} \, (DrinkingDecember \times Lockdown)_{ij} \\
&+ \beta_{18} \, (DryJanuary \times HolidayRelated)_{ij} + \beta_{19} \, (DrinkingDecember \times HolidayRelated)_{ij} \\
+u_{0j}^{(A)} + u_{1j}^{(A)} \, Weekend_{ij} + u_{2j}^{(A)} \, HolidayRelated_{ij} + u_{3j}^{(A)} \, AnnualSine_{ij} + u_{4j}^{(A)} \, DryJanuary_{ij} \\
&+ u_{5j}^{(A)} \, DrinkingDecember_{ij} + \varepsilon_{ij}.
\end{aligned}$$

Model 3. Overall daily alcohol consumption (linear mixed model; log scale)

$$\begin{aligned}
Y_{ij}^{(O)} &= \beta_0 + \beta_1 Weekend_{ij} + \beta_2 HolidayRelated_{ij} + \beta_3 Lockdown_{ij} + \beta_4 DryJanuary_{ij} \\
&+ \beta_5 DrinkingDecember_{ij} + \beta_6 AnnualSine_{ij} + \beta_7 AssessmentDay_{ij} \\
&+ \beta_8 AssessmentDay_{ij}^2 + \beta_9 AssessmentDay_{ij}^3 + \beta_{10} Gender_j + \beta_{11} Age_j \\
&+ \beta_{12}(Gender_j \times Age_j) + \beta_{13}(Weekend_{ij} \times HolidayRelated_{ij}) \\
&+ \beta_{14}(Weekend_{ij} \times Age_j) + \beta_{15}(Weekend_{ij} \times Lockdown_{ij}) \\
&+ \beta_{16}(DryJanuary_{ij} \times Lockdown_{ij}) + \beta_{17}(DrinkingDecember_{ij} \times Lockdown_{ij}) \\
&+ \beta_{18}(DryJanuary_{ij} \times HolidayRelated_{ij}) \\
&+ \beta_{19}(DrinkingDecember_{ij} \times HolidayRelated_{ij}) + u_{0j}^{(O)} + u_{1j}^{(O)} Weekend_{ij} \\
&+ u_{2j}^{(O)} HolidayRelated_{ij} + u_{3j}^{(O)} AnnualSine_{ij} + u_{4j}^{(O)} DryJanuary_{ij} \\
&+ u_{5j}^{(O)} DrinkingDecember_{ij} + \varepsilon_{ij}.
\end{aligned}$$

Overall daily alcohol consumption (overall AC) was modelled using a linear mixed model on the log scale; coefficients are reported on the log scale. Weekend indicates Friday–Saturday; holiday-related days include public holidays and the preceding day.

Random effects

Participant-specific random intercepts and random slopes for Weekend, Holiday-related day, Annual sine, Dry January, and Drinking December were included, with correlations among random effects. Final random-effects variance components and correlations are reported in eTable 2.

Estimation

Models were estimated in R (lme4 package) using maximum likelihood estimation with $\alpha = .05$. Missing EMA entries were handled in the mixed models, assuming data were missing at random conditional on observed covariates and random effects. No single or multiple imputation was applied. Detailed model specifications, including formula syntax, random-effects structures, and correlation assumptions, are fully documented in the rendered analysis scripts in the OSF repository [1].

eTables

eTable 1. Fixed effects for overall daily alcohol consumption (overall AC)

Overall AC				
Predictor	β	SE	z	*p* value
Intercept	2.5169	0.0450	55.98	**< .001**
Age (cohort)	0.0270	0.0030	9.06	**< .001**
Gender (men)	0.1675	0.0382	4.38	**< .001**
Weekend (Friday–Saturday vs other days)	0.2700	0.0196	13.76	**< .001**
Holiday-related day (vs other days)	0.2054	0.0216	9.51	**< .001**
Hard lockdown (vs other days)	0.0210	0.0169	1.24	.21
Annual pattern (sine)	0.1826	0.0185	9.89	**< .001**
January (Dry January)	-0.3230	0.0659	-4.90	**< .001**
December	0.7548	0.0435	17.34	**< .001**
Observation period, linear	-0.0003	0.0001	-2.63	**.009**
Observation period, quadratic	0.0000	0.0000	9.09	**< .001**
Observation period, cubic	-0.0000	0.0000	-7.21	**< .001**
Age × gender	0.0001	0.0029	0.04	.97
Weekend × hard lockdown	-0.0307	0.0107	-2.88	**.004**
Holiday-related day × weekend	-0.1174	0.0136	-8.63	**< .001**
Weekend × age	-0.0081	0.0009	-8.94	**< .001**
January × hard lockdown	0.0182	0.0377	0.48	.63
December × hard lockdown	0.1656	0.0301	5.51	**< .001**
January × holiday-related day	-0.1700	0.0539	-3.15	**.002**
December × holiday-related day	0.3080	0.0317	9.71	**< .001**

Note. SE = standard error; β = fixed-effect estimate. Overall daily alcohol consumption (overall AC) was modelled using a linear mixed model on the log scale; coefficients are reported on the log scale. Weekend indicates Friday–Saturday (vs all other days). Holiday-related days include public holidays and the preceding day. Hard lockdown contrasts hard-lockdown days (December 16, 2020, to February 28, 2021) with all other assessment days.

eTable 2. Random effects variance components and correlations of (a) Overall daily alcohol consumption (overall AC), (b) Drinking frequency (any vs none per day) and (c) Drinking-day amount (g/day on drinking days)

(a) Overall daily alcohol consumption (overall AC)

Random effect	Variance	SD	r (Weekend–Holiday)	r (Annual–January)	r (Annual–December)	r (January–December)
Intercept	0.819	0.905				
Weekend (Friday–Saturday vs other days)	0.0656	0.256				
Holiday-related day (vs other days)	0.0348	0.187	0.58			
Annual pattern (sine)	0.145	0.380				
January (Dry January)	0.519	0.721		0.29		
December	0.237	0.486			0.50	0.11
Residual	2.64	1.62				

(b) Drinking frequency (any vs none per day)

Random effect	Variance	SD	r (Weekend–Holiday)	r (Annual–January)	r (Annual–December)	r (January–December)
Intercept	2.35	1.53				
Weekend (Friday–Saturday vs other days)	0.0801	0.283				
Holiday-related day (vs other days)	0.0553	0.235	0.64			
Annual pattern (sine)	0.343	0.586				
January (Dry January)	1.21	1.10		0.32		
December	0.469	0.685			0.50	0.09

(c) Drinking-day amount (g/day on drinking days)

Random effect	Variance	SD	r (Weekend–Holiday)	r (Annual–January)	r (Annual–December)	r (January–December)
Intercept	0.166	0.407				
Weekend (Friday–Saturday vs other days)	0.0077	0.0876				
Holiday-related day (vs other days)	0.0071	0.0844	0.53			
Annual pattern (sine)	0.0214	0.146				
January (Dry January)	0.0214	0.146		0.18		
December	0.0285	0.169			0.29	0.28
Residual	0.402	0.634				

Note. Correlations are shown only for prespecified pairs (as referenced in the main text); blank cells indicate correlations not reported. Variance components and SDs are reported on the model scale. For the drinking-frequency (logit) model, residual variance is not estimated. Values are rounded for presentation (correlations to 2 decimal places; variance components and SDs to 2–4 decimal places depending on magnitude). Correlations are reported in the row of the second term in each pair (e.g., the Weekend-Holiday correlation is shown in the Holiday-related day row).

eFigures

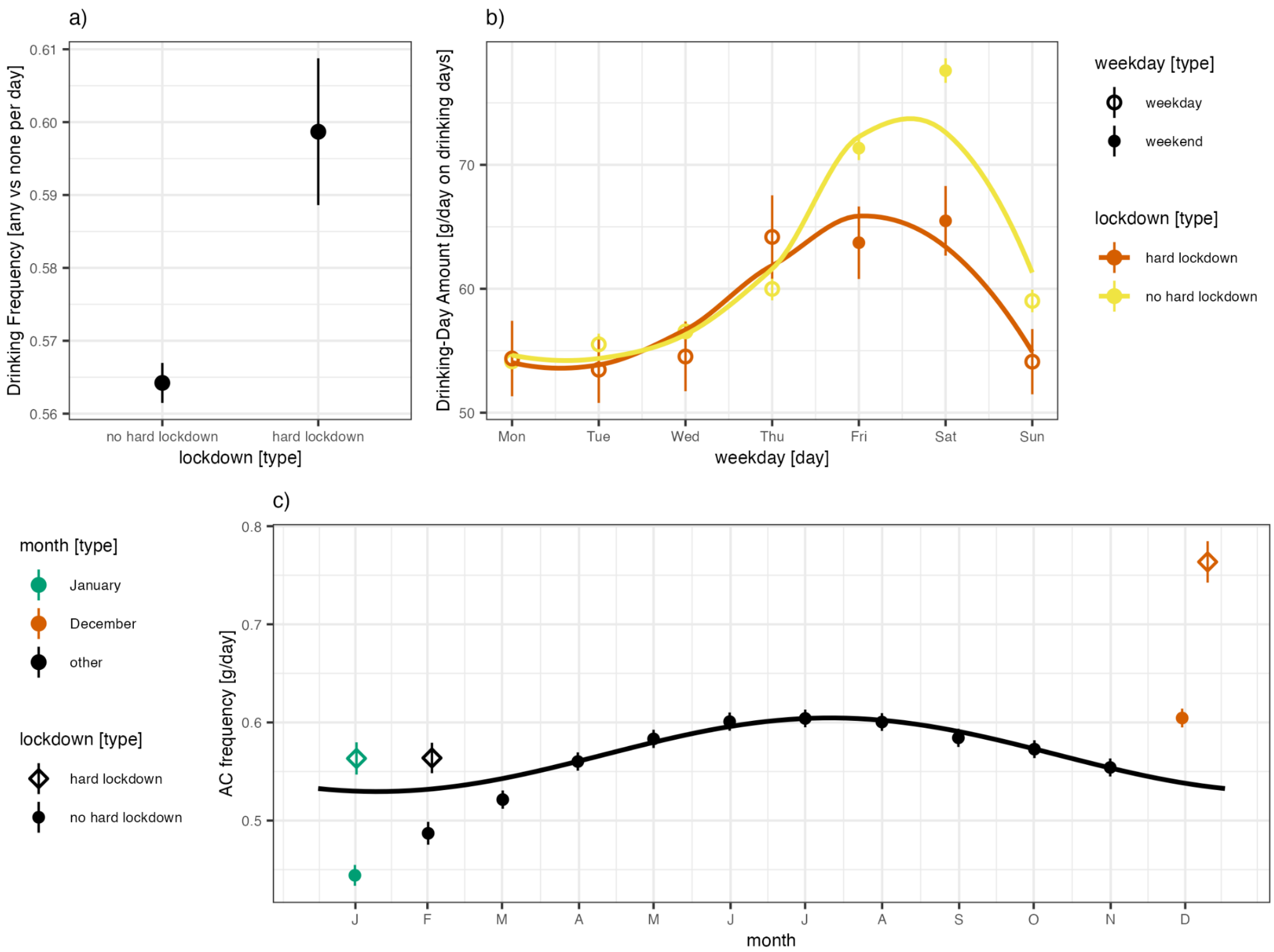

eFigure 1. Model-implied means of drinking outcomes by lockdown status and calendar time

a) Drinking frequency (any vs none per day) by lockdown type (hard lockdown vs no hard lockdown).
b) Day-of-week variation in drinking-day amount (g/day on drinking days) by lockdown type (hard lockdown vs no hard lockdown) and day type (weekday vs weekend).
c) Annual variation in overall daily alcohol consumption (overall AC; g/day) by month, highlighting January (Dry January) and December festive periods, stratified by lockdown type.
Note. Weekend indicates Friday–Saturday (vs all other days). Month categories indicate January, December, and other months. Points indicate model-implied means. AC indicates alcohol consumption.

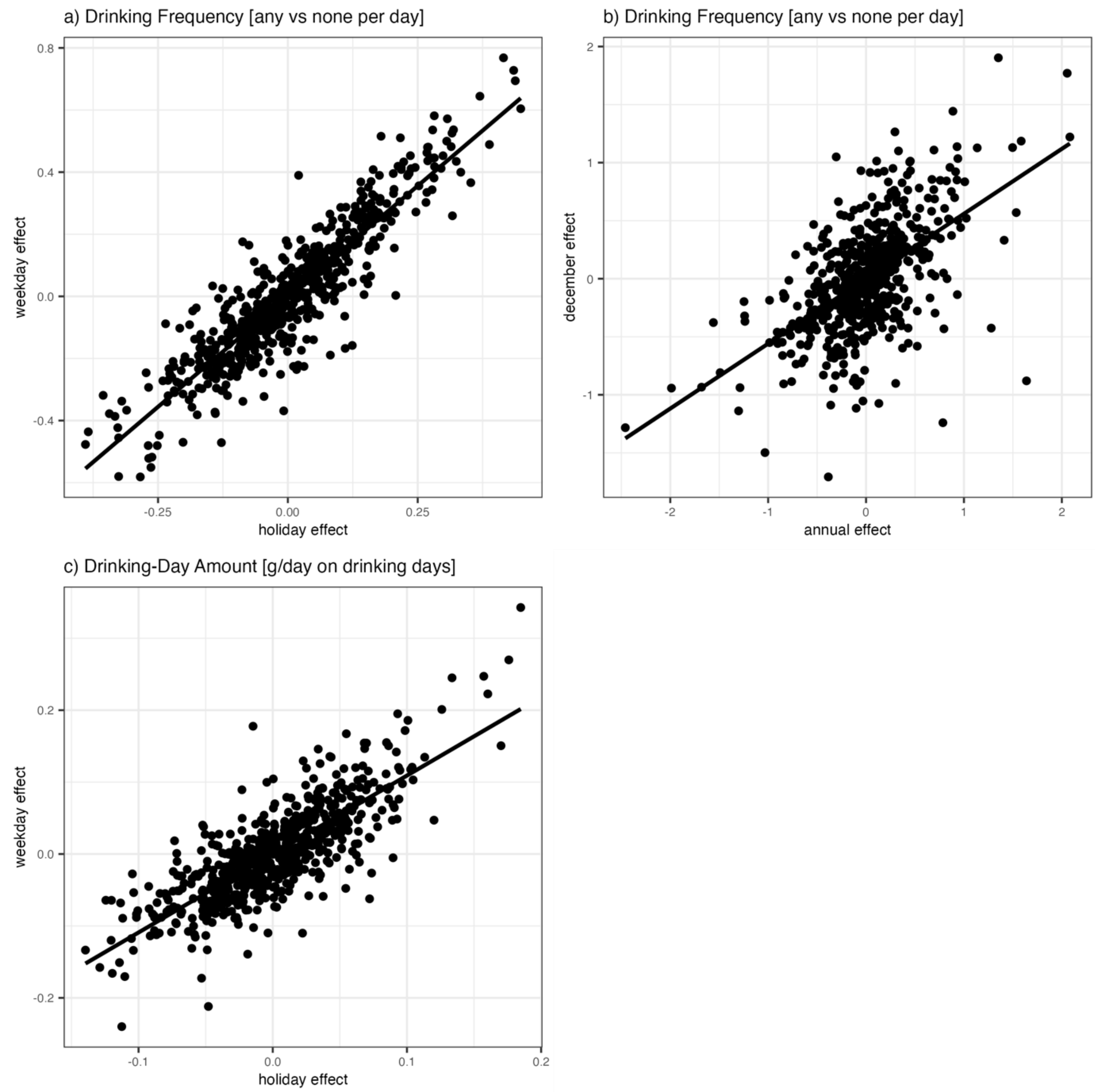

eFigure 2. Correlations between participant-specific random effects across temporal predictors

a) Association between individual-specific weekday-type (weekend vs weekday) and holiday-related day effects for drinking frequency (any vs none per day).
b) Association between individual-specific annual seasonal (sine) effects and december effects for drinking frequency (any vs none per day).
c) Association between individual-specific weekday-type (weekend vs weekday) and holiday-related day effects for drinking-day amount (g/day on drinking days).
Note. Each point represents a participant. Weekend indicates Friday–Saturday (vs all other days). Holiday-related days indicate public holidays and the day preceding a public holiday.

eReferences

1. Arntz F, Spitta G, Bahr NS, Rapp MA. OSF repository. Temporal patterns of alcohol use in individuals with AUD: A 12-month ecological momentary assessment study. 2026; https://osf.io/n3zph
